# Mechanisms of salinity tolerance and their possible application in the breeding of vegetables

**DOI:** 10.1186/s12870-023-04152-8

**Published:** 2023-03-14

**Authors:** Mostafakamal Shams, Ali Khadivi

**Affiliations:** 1 Department of Plant Physiology and Biotechnology, Faculty of Biology, University of Gdansk, Gdansk, Poland; 2 Department of Horticultural Sciences, Faculty of Agriculture and Natural Resources, Arak University, 38156-8-8349 Arak, Iran

**Keywords:** Salinity, Plant, Accumulation, Mechanisms

## Abstract

**Background:**

In dry and semi-arid areas, salinity is the most serious hazard to agriculture, which can affect plant growth and development adversely. Over-accumulation of Na^+^ in plant organs can cause an osmotic effect and an imbalance in nutrient uptake. However, its harmful impact can vary depending on genotype, period of exposure to stress, plant development stage, and concentration and content of salt. To overcome the unfavorable effect of salinity, plants have developed two kinds of tolerance strategies based on either minimizing the entrance of salts by the roots or administering their concentration and diffusion.

**Results:**

Having sufficient knowledge of Na^+^ accumulation mechanisms and an understanding of the function of genes involved in transport activity will present a new option to enhance the salinity tolerance of vegetables related to food security in arid regions. Considerable improvements in tolerance mechanisms can be employed for breeding vegetables with boosted yield performance under salt stress. A conventional breeding method demands exhaustive research work in crops, while new techniques of molecular breeding, such as cutting-edge molecular tools and CRISPR technology are now available in economically important vegetables and give a fair chance for the development of genetically modified organisms.

**Conclusions:**

Therefore, this review highlights the molecular mechanisms of salinity tolerance, various molecular methods of breeding, and many sources of genetic variation for inducing tolerance to salinity stress.

## Background

Salt stress is one of the main environmental factors reducing yield in arid and semi-arid areas where the irrigation water is saline and the rainfall is low. In other words, the accumulation of chlorides of sodium, calcium, magnesium, sulfates, and carbonates in soil solution can be defined as a climatic threat. Salt-impacted soils are varied in their physical and chemical characteristics. Hence, they vary in salt content, salt type, pH of the soil, temperature, distribution of salts in the soil profile, and the content of clay. Under such inconsistent situations, only a few plant species that are not susceptible occur naturally [1–3].

Regarding the hazardous effect of salinity, high levels of salinity can cause modifications in the plants’ performance, including the morphological, physiological, biochemical, and molecular functions characters. It changes photosynthetic activities, such as stomatal conductance, carboxylation, the substomatal concentration of CO_2_ (Ci), and the maximum quantum yield of photosystem II (PSII). Ultimately, it destabilizes the structure and permeability of the cell membrane and, the structure and function of the proteins, causing cell death [4, 5].

Vegetable crops are one of the main sources of natural antioxidants, such as various pigments, phenolic acids, and flavonoids [2, 6]. These are not just crucial sources of natural antioxidants but may also donate to human health due to the existence of various minerals, dietary fibers, and vitamins. Nonetheless, vegetables show various reactions to salt stress. Some vegetables, such as *Asparagus officinalis*, are tolerant to salinity stress, but some crops, such as bean (*Phaseolus vulgaris*), carrot (*Daucus carota*), and onion (*Allium cepa*) are susceptible to salinity stress. Accordingly, knowledge of the genetic basis of salinity tolerance in vegetables is a prerequisite for plant breeders to develop superior genotypes by adopting the traditional breeding procedure. In view of the truth that there is no single mechanism by which tension can be ameliorated, this review shall focus on salt tension, especially in terms of genomics. An attempt has been made to discuss the vision of salinity tolerance, the adaptive strategy, characteristics conferring salinity tolerance, and their use in conventional/traditional breeding activities for vegetable improvement.

## Impact of salinity stress on the plants

The response of plants to salinity is complicated, and salinity can damage plants via osmotic stress and ion toxicity, nutrient uptake balance, hormonal imbalance, and activities of antioxidants [6, 7]. The first response of physiological traits against salinity stress is the stomatal closing and a decline in leaf extension. In the lower leaves of plants, we can find the over-accumulation of Na^+^ and Cl^−^ and its toxic impact on ions concentration. Consequently, the accumulation of salts and imbalance in nutrient uptake leads to a decrease in the leaf area index [8, 9].

Additionally, in sodic soils, hyperosmotic pressure causes the cells to lose more water than they take in. Water deficiencies cause a chain reaction of physical, signaling, gene expression, metabolic, and physiological pathways and activities, leading to a decrease in leaf area index, photosynthesis, and biomass accumulation and, eventually, reducing yield [2, 10–12]. In sodic soils, NaCl includes 50–80% of soluble salts, and this is the main cause of increasing the concentration of Na^+^ and Cl^−^ ions and toxicity in plants [13, 14]. These ions impact the biochemical process of enzyme activities or the signaling pathway of photosynthetic activities significantly [3, 15]. Also, due to physical, chemical, and transport system interactions between Na^+^ and K^+^, the Na^+^ in the solution of saline soils competes with K^+^ for absorption and can lead to the deficiency of K^+^ in plant tissues [7, 16, 17]. The conducted deficiency in K^+^ concentration in tissues especially in leaves minimizes growth since it performs an essential role in photosynthetic parameters, such as stomatal conductance and function, and regulation of cell turgor [18, 19].

Photosynthesis in vegetables is a complex plant-physiological and biochemical mechanism that is influenced by salt tension extremely [3, 20]. Stomata closure can be conducted in a decrease in carboxylation and photosynthesis efficiency in salt-sensitive vegetables. Salinity stress also impacts photosynthesis by chlorophyll degradation through overexpression of pheophorbide *a* oxygenase (*CaPAO*) [3]. Furthermore, salinity can cause disorders in PSII function and oxygen-evolving complex, and cyclic electron transport [4, 5]. In parallel, it was documented that salinity stress reduced transpiration rates and stomatal conductance, and caused to degradation of pigments and light-harvesting complexes, and as result, it led to a reduction in quantum yield of photosynthetic, and dissipation of energy by non-photochemical strategies in lettuce, onion, tomato, and sweet potato [20–23].

Regardless of the primary impacts of salt stress, the secondary effect of salinity on plant cells is accumulation of reactive oxygen species (ROS), which are toxic and cause DNA methylation and affect gene expression [24, 25]. Moreover, ROS can cause lipid peroxidation, which improves the membrane’s fluidity and permeability [26]. However, plants have developed extensive internal tolerance systems, such as enzymatic and non-enzymatic antioxidants to overcome the results of ROS accumulation [3, 27]. Salt tolerance of some vegetables is shown in Table 1.

**Table 1:** Salt tolerance of some vegetables

Sensitive	Moderately sensitive	Moderately tolerant	Tolerant
*Daucus carota*	*Brassica oleracea*	*Solanum lycopersicum*	*Asparagus officinalis*
*Raphanus sativus*	*Ipomoea batatas*	*Ocimum basilicum L.*	
*Pisum sativum*	*Curcuma longa*	*Beta vulgaris subsp. Vulgaris*	
*Solanum melongena*	*Allium sativum*	*Cucurbita pepo ‘Yellow scallop’*	
*Brassica rapa*	*Colocasia esculenta*		
*Allium cepa*	*Solanum tuberosum*		
*Abelmoschus esculentus*	*Cucurbita moschata*		
*Manihot esculenta*	*Citrullus lanatus*		
*Phaseolus vulgaris*	*Capsicum annuum*		

## Mechanism of salt tolerance

Some vegetable crops, such as *Beta vulgaris* have the natural capability of tolerating the adverse impacts of high NaCl in the root zone or on the leaves without adversely affecting their yield by having the highest leaf relative water content (LRWC), leaf area index, biomass accumulation, and photosynthesis. Some crops are mitigated the adverse impact of NaCl via minimizing the absorption of Na^+^ and Cl^−^ ions to the shoots, and osmotic adjustment. In the root, salts can penetrate root membranes through active or passive transport mechanisms. Ion path has been revealed to be selectively adjusted through plasmalemma, tonoplast, or specialized cells along with xylem parenchyma. Individual ion-specific ATP’s that adjust this ion activity can be impacted by the concentration of other ions. Consequently, a higher variation can be found among the genotypes in their capability in adjusting the concentration of ions and the production and accumulation of the osmotic substances in reaction to sodic conditions. Nonetheless, some organic acids and amino acids, such as malate, oxalate, glycine betaine, and proline have been identified as potential osmotic mechanisms in higher plants. Carbohydrates can also be selectively aggregated in response to drought and salinity stress [6, 7, and 28].

**Fig. 1:**
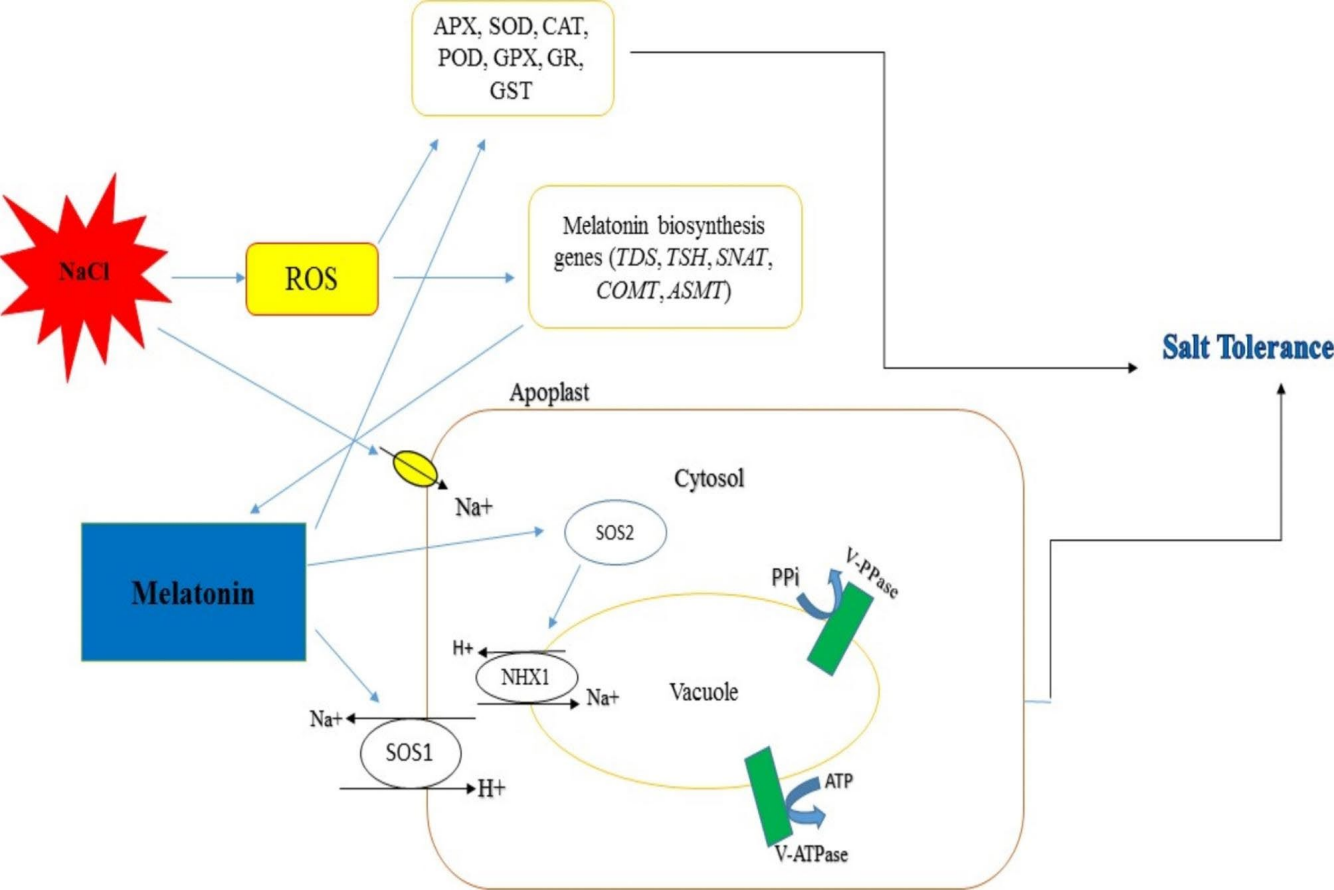
The suggested model depicts melatonin’s signaling function in conferring salt stress resistance in plants. By being exposed to salinity, Na^+^ produces ROS, which activates antioxidant and non-antioxidant enzymes as well as melatonin. Melatonin activates antioxidant and non-antioxidant enzymes, as well as, increases *SOS* and *NHX* activities. SOS transports excess Na^+^ to the extracellular space using the plasma membrane proton gradient. NHX on the vacuole tonoplast contributes to Na^+^ homeostasis (created using Inkscape software ver.1.2.2). Na^+^/H^+^ exchanger1 (*NHX1*); Salt overly sensitive1 (*SOS1*); Salt overly sensitive2 (*SOS2*); Ascorbate peroxidase (APX); Superoxide dismutase (SOD); Catalase (CAT); Peroxidase (POD); Glutathione peroxidase (GPX); Glutathione reductase (GR); Glutathione transferase (GST)

Melatonin, dopamine, and eATP are Neurotransmitters, which can adjust K^+^ and Ca^2+^ permeable ion channels and also plays a main function in ionic homeostasis under NaCl stress in plants. Among neurotransmitters, melatonin is isolated from tryptophan and can induce tolerance to salinity in plants by activating antioxidant systems and adjustment of the expression of K^+^ uptake (*AKT*), Na^+^/H^+^ exchanger (*NHX*), and salt overly sensitive (*SOS*) (Fig. 1), which are known as ion channels genes [29, 30]. In *Brassica*, the treatment of melatonin by activating *NHX1*, and *SOS2* improved vacuolar Na^+^ sequestration and Na^+^ exclusion for reducing Na^+^ toxicity under salt stress [29].

Dopamine, 3, 4-dihydroxyphenethylamineis, is delivered from tyrosine amino acid in plants and is a non-protein amino acid that belongs to the families of catecholamine and phenethylamine. Under salinity stress, dopamine can also enable vegetables, such as *Brassica* species to overcome osmotic stress through sensing osmotic pressure alterations by adjusting or triggering mechano-sensitive cation channels. Notably, the mentioned channels may be practicable candidates for discerning toxicity or deficiency of ions in plant organs, as they could alter mechanical modifications influenced by turgor pressure into electrical signals [9, 29, and 31].

Besides the above-mentioned mechanism, under adverse conditions, plants evolved antioxidant defense systems that used ROS-scavenging processes. Antioxidant enzymes, including superoxide dismutase (SOD), peroxidase (POD), and catalase (CAT) are employed to relieve ROS to maintain redox homeostasis under salinity stress [9]. Furthermore, salinity-induced osmotic stress stimulates the synthesis of the phytohormone abscisic acid (ABA), which controls plant physiological activities in response to diverse abiotic stress. A higher level of salinity can stimulate ABA synthesis in plants by triggering the expression of biosynthesis pathway genes. ABA not only transmits salinity signals to cells, but it also protects them from salt stress by altering the expression of stress-responsive genes [29].

Plants, nonetheless, respond to and adjust to different environmental factors via morphological, anatomical, and biological acclimation at the cellular and plant levels. These morphological, anatomical, and physiological adaptations assist the plant in coping with environmental stress. As is widely known, the anatomical flexibility of leaf and root in plants is vital in conferring tolerance under numerous abiotic stresses, such as salinity and drought stresses [30]. An enhancement in both leaf cuticle and leaf epidermal thickness is one of the anatomical traits in salt-tolerant genotypes in general. Also, physiological traits, such as low photosynthesis, closed stomata, high leaf temperature, and a decrease in leaf turgor and chlorophyll content are well-known physiological mechanisms for tolerating salinity stress in vegetables. Furthermore, intact bulliform cells, bulliform cell area, chloroplast content, and chloroplast ultrastructure, particularly the length, width, and width/length of chloroplasts, can be efficient indicators for salt-unsusceptible genotypes in monocotyledon vegetables, such as sweet corn, asparagus, yams, leeks, and onions [7].

## Genetic mechanism of salinity tolerance

The next generation of sequencing, the technology of high-throughput sequencing technology, offered the way for the whole genome sequencing of vegetables. Because of the high capability of sequencing and decrease in sequencing costs, it has led to the discovery of multiple molecular strategies in abiotic tension and therewith contributed to the elaboration of functional genomics of tolerance to abiotic stress, such as salinity in vegetables [32]. Nonetheless, because of the heterozygous nature of most vegetables, and also polyploidy, diversity, and complexity, sequencing of whole genome has been completed in a little number of crops. Also, most of the quantitative potential characters in the vegetables are polygenic traits controlled by numerous alleles/genes located across the entire genome [33]. Due to the forenamed limitations, we can find a shortage of genome information and varied molecular strategies for tolerance to abiotic stress in vegetables. Hence, next-generation sequencing must be done through the whole genome of important vegetables. Recently, advanced bioinformatics methods and genotyping-by-sequencing (GBS) data have offered novel genetic resources of tolerance to environmental stress in vegetables [34]. Because of this, we can find the development of various molecular markers, such as SNP (single nucleotide polymorphism), SSR (simple sequence repeats), and RNA-based molecular markers. Through them, the election cycle has been shortened, resulting in the development of genetic gain, mapping for quantitative trait locus (QTLs) related to tolerance to abiotic stress, and identification of abiotic characters. Among vegetables, reference genomes for various Solanaceae and Cucurbitaceae genotypes have been sequenced as well as assembled, such as tomatoes, peppers, cucumbers, watermelons, and melons. In tomatoes, various association mapping studies, such as genome-wide association studies (GWAS), have been performed for fruit traits, volatiles, and abiotic/biotic tolerance [35, 36]. Genome sequencing also revealed more than 22,000 candidate regions across the whole genome that could act to develop different SSR and SNP markers in *Dioscorea rotundata* (white guinea yam). Under salinity stress conditions, silico gene expression analysis of the transcriptomic profile identified 6,233 SSR markers in *Ipomoea imperati* [37]. These markers are valuable for comparative genomics research and indirect selection in the development of salt tolerance in sweet potatoes [38–40]. Nonetheless, Liu et al. [41] assessed the mechanisms of miRNA-mediated gene adjusting in grafted watermelon under normal conditions. They also identified the expression of 47 miRNAs in watermelon grafted on Shintozwa’ (*Cucurbita maxima*×*Cucurbita moschata*) and 20 miRNAs in the ones grafted on *Lagenaria sicerariawere* significantly, relative to self-grafted watermelon. Nonetheless, Ram et al. [42] identified 31 putative *TLP* gene families in watermelon and named them *ClaTLP1* to *ClaTLP31*, and this gene family is distributed randomly on some chromosomes rather than covering of whole genome of watermelon under salinity and drought stresses. Chromosome 10 represented the maximum number of *ClaTLP* genes. Out of 31 gene families, five *ClaTLP* genes (16%) showed tandem duplication whereas only 4 genes (12.90%) resided in segmentally duplicated blocks on watermelon chromosomes.

However, there are few reports on vegetables that use genomic resources to develop lines with salt-tolerant traits. Accordingly, future studies must focus on QTL mapping to discover characters and genes for salt tolerance. GWAS, and sequencing can also accelerate breeding by shortening the breeding cycle, as well as discover salt-tolerant functional genotypes by re-sequencing. As a result, manipulating these sensitive genes and cross-transfer between genotypes will be critical in instilling tolerance to salinity and comprehending the underlying molecular strategies.

Nonetheless, polyploidy is an effective strategy to improve crop abiotic stress tolerance by affecting morphological and anatomic traits, as well as physiology, biochemistry, and gene expression. Many plants that are currently grown and exploited for world food are also polyploidy. In the case of polyploidy induction, it is a procedure that gives the plant breeder the opportunity to modify a plant by altering the number of chromosomes and, consequently, the proportion of allelic genes that contribute to the appearance of characters. Also, polyploidy plants can tolerate abiotic stress better than diploid genotypes. For instance, polyploidization of *Lettuce sativa* var. *crespa* promoted chromosomal duplication in originally diploid lettuce seedlings (2n = 18), and it was discovered that polyploidy-induced seedlings had a higher dry weight and leaf area per seedling under saline stress [43].

## Transcriptomics

Advancements in the capability of next-generation sequencing techniques and a decrease in sequencing costs have precipitated transcriptomic studies on abiotic and biotic stress in crops. The primary function of this technique is that it decodes various transcripts or genes which have a significant function in imparting tolerance to salinity in crops [44]. Exposing plants to adverse conditions, such as salinity is associated with the upregulation or downregulation of various genes with many functions, which can cause a resultant modification in the process of plant growth and development and can facilitate tolerance to stress [45, 46]. Table 2 shows potential candidate genes responsible for tolerance to salt stress in various vegetable crops.

**Table 2:** Candidate genes responsible for salt tolerance in vegetables

Common name	Species	Gene name	Gene function	Reference
Tomato	*Solanum lycopersicum*	*BADH-1*	Overexpression of betaine aldehyde dehydrogenase	[84]
	*Solanum lycopersicum*	*NHX1*	Over expression the NHX1 antiporter	[84]
	*Solanum lycopersicum*	*VP1.1*	Increased proton pumping	[84]
	*Solanum lycopersicum*	*TPS1*	Synthesis of compatable solotus	[84]
	*Solanum lycopersicum*	*APX6*	Degradation of reactive oxygen species	[84]
	*Solanum lycopersicum*	*NHX2*	Na+/H + antiporter 2	[85]
	*Solanum lycopersicum*	*bZIP1*	bZIP transcription factor6	[86]
	*Solanum lycopersicum*	*JUB1*	Increasing protein level	[87]
	*Solanum lycopersicum*	*SlGMEs*	Ascorbate accumulation	[88]
	*Solanum lycopersicum*	*NRT2*	nitrate transport	[89]
Cucumber	*Cucumis sativus*	*CsPP2-A1*	participating ABA-JA signaling pathway and antioxidant system	[90]
	*Cucumis sativus*	*CsPNG1*	deglycosylating misfolded glycoproteins	[101]
	*Cucumis sativus*	*CsASR1*	protect the activity of lactate dehydrogenase	[92]
Watermelon	*Citrullus lanatus*	*ClWRKYs*	Growth, development, biotic and abiotic stress response	[93]
Lettuce	*Lactuca sativa*	*MAPK*	Coline oxidase	[94]
Muskmelon	*Cucumis melo*	*MATE*	Vacuolar Na+/H + antiporter	[95]
Carrot	*Daucus carota*	*DcPSY*	Biosynthesis of carotenoids	[96]
	*Daucus carota*	*Carrot WRKYs*	Hormone and mechanical injuries	[97]
Eggplant	*Solanum melongena*	*SmCBF*	Salinity tolerance	[98]
	*Solanum melongena*	*ARF*	Salinity tolerance	[99]
	*Solanum melongena*	*POD*	Peroixidase	[100]
Pepper	*Capssicum annuum*	*CaATG8c*	autophagy activities	[3]
Sweet potato	*Ipomoea batatas*	*IbNHX2*	Na+/H + antiporter	[102]
Sugar beet	*Beta vulgaris*	*cystatin gene*	cysteine protease	[102]
Sugar beet	*Beta vulgaris*	*BvM14-SAMS2*	S-Adenosyl-l-Methionine synthetase	[103]
Onion	*Allium cepa*	*PSII gene*	Photosystem II	[104]
		*CAT*	Catalase	[104]

It was documented that many genes encode regulative proteins, such as TFs, which adjust the pathways of salt sensing and transduction of signals and the expression of a range of salinity stress-reactive genes, while some of these up-regulated or down-regulated genes code proteins that act as the main function in stress-related growth or metabolic changes [1, 47].

In a previous study, in the *Cucumis melo* L. genome, 82 genes of NAC (*CmNAC*) were discovered [48]. In that study, the researchers identified that *CmNAC* genes are implicated significantly in the response to salinity stress. Also, the expression analysis demonstrated that under salinity stress, about 12 genes of *CmNAC* genes were overexpressed in the roots of melon and they induced tolerance to salinity in melon. In a study, the WRKY transcription factor of sweet potato was cloned in Arabidopsis and the transgenic seedlings of Arabidopsis showed tolerance to salinity and drought stress [49]. In pepper, Zhang et al. [46] demonstrated that *CaNAC035* is affected by abiotic stress, such as salinity. To comprehend the function of *CaNAC035* under abiotic stress, they applied virus-induced gene silencing in pepper to observe the knockdown and overexpression of *CaNAC035* in pepper and Arabidopsis, respectively. Their results revealed that seedlings of pepper in which *CaNAC035* was silenced, exhibited more sensitivity than the control groups under salinity stress. Also, lower chlorophyll content was found in *CaNAC035*-silenced seedlings, but a higher symptom of tolerance to salinity stress was found in *CaNAC035*-overexpressed Arabidopsis. They also identified 18 proteins that potentially interact with *CaNAC035* and can partake in functions, such as response to salinity stress, tolerance, and photosynthetic activities. Their results indicated that *CaNAC035* is a positive controller of tolerance to abiotic tension in capsicum, which performs via various signaling pathways.

Apart from NAC transcriptome factors, the responsible genes for cellular homeostasis, phytohormone modulation, and osmotic adjustment perform the main functions in deriving tolerance to environmental stress [48]. In the transgenic seedlings of *Solanum lycopersicum* L. cv. MicroTom overexpression of *LeNHX2* and *SlSOS2* genes led to overexpression of K^+^, Na^+^/H^+^ antiporter, and the regulatory kinase *SlSOS2* under salinity stress. All transgenic plants demonstrate better tolerance to salinity than wild-type and the seedlings overexpressing both *SlSOS2* and *LeNHX2* outperformed those overexpressing only one of these genes in terms of plant yield. Furthermore, higher K^+^ content was observed in the fruits of all transgenic plants relative to the wild type, and also higher Na^+^ content was observed in the fruits of plants that expressed *SlSOS2* under excessive salinity stress. The transgenic seedlings overexpressing *LeNHX2* had higher K^+^ levels in their leaves, stems, and roots than wild-type and single transgenic seedlings overexpressing *SlSOS2*. The plants with *SlSOS2* overexpressing grown under salinity had a higher content of Na^+^ in stems and leaves compared to the wild-type and *LeNHX2* single transgenic plants. A higher LRWC and water use efficiency were observed in all transgenic lines than in wild-type (control) under salt conditions. The results of this study suggest that the combined overexpression of *LeNHX2* and *SlSOS2* enhances growth and LRWC under salinity stress, and also impacts K^+^ and Na^+^ homeostasis, and improves the yield of tomato plants [50].

Genes of the calcium signaling pathway and the pathway of mitogen-activated protein kinase (MAPKs) play a crucial function in tolerance to salinity by triggering salinity-responsive genes. For potatoes, the genes of the Ca^+^ signaling pathway (CBLs) and *MAPKs* signaling pathway genes (*MAPKK9, MAPK4*, and *MAPK6*), were overexpressed in salinity-tolerant cultivars, illustrating their function under salinity [51]. Similarly, RNA sequencing analysis revealed that *WRKY22* and other hormone signal transduction pathways perform a significant function in providing tolerance to salinity stress in the tolerant genotype of wild-type sweet potato (*Ipomoea pescaprae*) [52].

Additionally, transcriptome analysis demonstrated that the genes of the SOS pathway (*SOS2*, NHX protein genes, V-ATPase genes, and PM-ATPase genes) play an important role in the growth and performance of potato under salinity stress. Also, it was shown that in sweet potato, jasmonic acid plays a crucial performance under salinity conditions. Under 200 mM NaCl, transcriptome analysis indicated that jasmonic acid biosynthesis and signaling genes, including *IbLOX, IbAOS, IbAOC, IbOPR3, IbCOI1*, and *IbPDF1.2* were overexpressed in the salinity-tolerant line of sweet potato [46]. Also, it was demonstrated that genes of ABA signaling pathways (*PYL*, *SnRK2*, and *MPK* kinase) were up-regulated in tomato demonstrating their role in inducing tolerance to salinity [53].

Heat shock proteins (HSPs) accumulate under abiotic/biotic stress in plants. The autophagy of non-useful proteins is done by HSPs under salinity and drought stresses. Watermelon is susceptible to environmental stresses and, in a previous study, the role of HSP20 proteins was explored in watermelon. In this respect, the qRT-PCR analysis and GUS staining demonstrated that exogenous treatment of ABA, and salinity stress markedly decreased the expression of *ClHSP22.8* (a member of the HSP20 family). However, in transgenic lines of Arabidopsis, the over-expression of *ClHSP22.8* caused hypersensitivity to ABA, and tolerance reduction to salinity stress. The results showed that overexpression of *ClHSP22.8* has a negative role in the plant defense mechanism system under salinity stress [54].

The comparison and analysis of transcriptome data in salt-tolerant and salt-susceptible genotypes in vegetables can make very evident the function of the signaling pathway, the integrity of the membrane, transcription factors, and the synthesis of phytohormones. Thus, these are potential targets for breeding salinity-tolerant genotypes in vegetable crops.

## Breeding for salinity tolerance: conventional and modern approaches

To develop salinity tolerance genotypes, smart breeding is important in vegetables [55, 56]. Sequencing of whole genome plays a significant role in the genomic study, proteomics, transcriptomics, metabolomics, and QTLs studies for increasing tolerance to salinity in vegetables. Collecting germplasm, including wild genotypes has supplied significant genetic diversity for tolerance to salt, and the prosperity of breeding techniques relies mainly on the germplasm selections. Screening of plant germplasms to identify salinity-tolerant genotypes has accelerated the production of salinity-resistant cultivars through gene transfer [34, 57].

In conventional breeding methods, the breeders have applied hybridization, recurrent selection, pedigree selection, backcrossing, and induced mutation approaches for developing salinity-tolerant genotypes in vegetables. However, new approaches to breeding, such as CRISPR/Cas9, CRISPR/Cpf1, prime and base editing, dCas9 epigenetic changes, and various further transgene-free genome editing approaches exist to supply the lacuna of selection cycles and the restricted genetic diversity [58].

Additionally, polyploidy can play a vital role in species invasion success through a combined application of (1) ‘pre-adaptation’, whereas polyploid lineages are primed to conditions in the new range and, consequently, have higher rates of survival and fitness in the earliest establishment stage; and (2) the possibility for successive acclimation due to a larger genetic variation that may assist the ‘evolving of invasiveness’. Polyploidization, on the other hand, may play a significant role in (3) restoring sexual reproduction after hybridization or, conversely, (4) asexual reproduction in the absence of appropriate individuals [59]. Polyploidy is an important evolutionary factor in both wild and domesticated plants. Polyploid organisms frequently have more vigor and, in certain situations, exceed their diploid relative in a variety of ways. Polyploids’ exceptional superiority has been the goal of many plant breeders over the last century, who have induced polyploidy and/or utilized natural polyploids in a variety of methods to produce ever-enhanced plant cultivars [60]. The expansion of plant organs (“gigas” impact), buffering of harmful mutations, increased heterozygosity, and heterosis are some of the most significant implications of polyploidy for plant breeding (hybrid vigor). In terms of this technique, the genotypes with high performance and growth, and increased resistance to both biotic and abiotic stressors have been developed. When crossing between two species is not possible due to ploidy level discrepancies, polyploids can be employed as a bridge for gene transfer between them. Furthermore, polyploidy frequently causes lower fertility due to meiotic mistakes, permitting the development of seedless cultivars. In contrast, the genome doubling in a newly generated sterile hybrid offers the recovery of its fertility [60].

Nonetheless, the MAS is known as a molecular marker related to the agronomics character of interest controlled by single genes or QTL. This is the maximum efficient and effective way to create new salinity-tolerant vegetable genotypes. With the application of MAS, the breeding process can be shortened by screening crops with the target genes at earlier growth stages, saving time and material, and agronomic inputs, such as fertilizers, herbicides, and irrigation, and abiotic factors have no effect.

For salinity tolerance to introgress/transfer-related genes, marker-assisted backcross (MABC) breeding is a more accurate and efficient technique. It allows for plant selection throughout each breeding season to ensure the presence of introgressed target genes, as well as the restoration of the parental genome (RPG) and decreased linkage drag [61–63]. To specify different QTLs and the introgression of Quantitative characters in vegetables by MAS, several molecular markers are accessible. Various DNA markers, including SNP, restriction fragment length polymorphisms (RFLP), amplified fragment length polymorphisms (RFLP), sequence-tagged sites (STS), and SSR, can be utilized for genetic analysis in molecular identification and characterization and MAS [64, 65]. GWAS was used to discover the Or gene on chromosome 3, as well as the link between terpenoid biosynthesis pathway genes and volatile terpenoid odor ingredients in carrot [66, 67]. Whether candidate genes were specified using GWAS in various germplasm collections or bi-parental populations, carrots were screened for salt tolerance using plant introductions (wild germplasms and commercialized hybrids) [68], and five developed carrot genotypes (PI 509,433, PI 652,374, PI 652,402, PI 652,403, and PI 652,405) were shown to have greater tolerance to saline.

Kompetitive Allele-Specific PCR (KASP™) is a novel and fast genotyping method of SNP genotyping which needs only a few markers of SNP to genotype different samples. In a previous study, the genetic variety of 73 onion genotypes, including wild type, commercial, and native variants, from around the world was investigated using KASP genotyping technique. With the evaluation of The SNP dataset, 375 polymorphic loci with a very low percentage of non-calling sites (0.03%) were retrieved [68]. 89% of the genotypes of onions amplified all polymorphic loci and were contemplated for structural analysis of the population. The ΔK approach allowed for the discovery of gene pools and proposed four populations, each representing the geographical source of the genotypes. Based on their statistical analysis, their SNP set was successful in introducing potential genotypes for use in successful breeding projects and revealing population-specific alleles. It is worth noting that seventy-four loci were linked with phenotypical characteristics, such as bulbing photoperiod, bulb shape, or bulb color, and tree loci were specified as potential selections target for onion improvements [69].

A wild type of tomato (*Solanum pimpinellifolium*) presents a breeding potential source for desired features, including abiotic and biotic stress tolerance. The genome sequencing of *S. pimpinellifolium* ‘LA0480’ was performed by Razali et al. [70]. Furthermore, they presented phenotypic results from a field study that showed *S. pimpinellifolium* had a high tolerance to salinity relative to cultivated tomato. The results showed that annotated gene numbers (25,970) and the ‘LA0480’ genome assembly size (811 Mb) were within the range identified for other sequenced tomato genotypes. Additionally, they advanced and used the Dragon Eukaryotic Analysis (DEAP) platform to functionally annotate genes encoding the ‘LA0480’ protein. Their findings revealed that genes involved in biotic and abiotic stress responses were overrepresented. For understanding the genetic basis for such variations in *S. pimpinellifolium* and *S. lycopersicum*, they explored fifteen genes that have formerly been demonstrated to promote tolerance to salinity. They discovered that *S. pimpinellifolium* had higher homologs of the inositol-3-phosphate synthase and phosphatase genes, which are both key enzymes in the biosynthesis of inositol and its byproducts. Nonetheless, their findings suggested that changes in the inositol phosphate chain may lead to greater salt tolerance [70].

CRISPR is a gene-editing technology commonly utilized in numerous species. The desired plant phenotype has been achieved in numerous crops by specific genetic tinkering utilizing CRISPR-CAS systems [71, 72]. The CRISPR/Cas9 technique consists of a single guide RNA (sgRNA) molecule with a specific target sequence that is homologous to a target gene and the DNA endonuclease Cas9. The utilization of the CRISPR/Cas9 system necessitates the construction of a reliable and ubiquitous technique for transferring CRISPR/Cas9 reagents in cells [73]. Effective delivery techniques involve protoplast transfection, Agrobacterium (*Agrobacterium tumefaciens*)-mediated transformation, and particle bombardment [74–77]. The most extensively utilized delivery strategy for producing crop varieties and achieving successful targeted mutagenesis is A*grobacterium*-mediated transformation [77]. CRISPR/Cas9 success has been documented in Arabidopsis and several significant vegetables, such as a tomato (*Solanum lycopersicum*), broccoli (*Brassica oleracea* var. *italic*), melon (*Cucumis melo*), lettuce, and carrot [78–82].

The genes of auxin response factors (ARF) perform a key function in plant responses to environmental stresses. Both OsARF11 and OsARF15 are expressed differentially in plants under salt stress conditions [83]. A CRISPR/Cas9-induced SlARF4 mutant showed tolerance to salinity compared to the control [78].

## Conclusion

As reported by different studies, vegetables have varying degrees of tolerance and susceptibility to salt. Today, salt is a major issue for food security since it reduces the quality and quantity of vegetables during their growth and development stages. Salinity causes osmotic, water, and nutrient imbalances, as well as cellular interference and morphological interference, which eventually impact plant physiochemical processes. Tolerance to salt is the outcome of a complex system, including phytohormones, antioxidants, phytoprotectants, and the co-regulation of numerous genes and their relationships. While it is a complex mechanism, numerous genes related to tolerance in some genotypes of vegetables were identified, and salinity-tolerant genotypes can be generated by transferring them via breeding methods into susceptible genotypes. However, a conventional breeding method demands exhaustive research work on vegetables and depends on mixing characteristics from different populations within a species and then selecting from the plant’s natural complement of genetic elements. Therefore, new techniques of molecular breeding, such as cutting-edge molecular tools, and CRISPR technology are also now available in economically important vegetables and give fair chance for the development of genetically modified organisms (GMOs).

## Data Availability

Not applicable.
